# Vascular Changes in Bleomycin-Induced Scleroderma

**DOI:** 10.1155/2011/270938

**Published:** 2011-10-19

**Authors:** Toshiyuki Yamamoto, Ichiro Katayama

**Affiliations:** ^1^Department of Dermatology, Fukushima Medical University, Hikarigaoka 1, Fukushima 960-1295, Japan; ^2^Department of Dermatology, Osaka University, Yamadaoka 2-2, Suita, Osaka 565-0871, Japan

## Abstract

Systemic sclerosis (SSc) is characterized by vascular injury, immunological abnormalities, and fibrosis of the skin as well as various internal organs. Vascular impairment is the early manifestation and plays a fundamental role in the pathogenesis of SSc. Recent studies suggest that complex interactions among the endothelial cells, pericytes, smooth muscle cells, and fibroblasts are involved in the systemic vasculopathy in SSc, and histological feature of proliferation of vascular wall is seen in the lesional scleroderma skin at the late stage of disease. One of the most representative mouse models for scleroderma is the bleomycin-induced scleroderma; however, aspects of vascular alteration have not been described in detail so far. A number of studies have shown that bleomycin stimulates endothelial cells and fibroblasts to induce proinflammatory and fibrogenic cytokines, apoptosis, reactive oxygen species, and so on. This paper makes a focus on the vascular involvement in the bleomycin-induced murine scleroderma.

## 1. Introduction

Systemic sclerosis (SSc) is a connective tissue disease which shows fibrosis of the skin and various internal organs [1]. Although the pathogenesis of SSc has not been fully elucidated yet, it is characterized by vascular injury, immunological abnormalities, and excessive accumulation of extracellular matrix (ECM) proteins in the skin and various internal organs. In particular, systemic vasculopathy plays a fundamental role in SSc and is associated with various altered vascular dysfunctions in the lung, kidney, heart, and skin. Clinically, Raynaud's phenomenon, digital ulcers, and abnormal nailfold capillaries are seen in association with peripheral vasculopathy. Raynaud's phenomenon is caused by vasospasm, commonly seen in patients prior to the onset of sclerodactyly. Endothelial cells have been reported to play an important role in the initial inflammatory as well as subsequent fibrotic process. Histological analysis of the initial stage of scleroderma reveals perivascular infiltrates of mononuclear cells in the dermis, which is associated with increased collagen synthesis in the surrounding fibroblasts. T-cell interaction with vascular endothelial cells may lead to the subsequent cellular immune reaction, which may induce further vascular injury and tissue fibrosis. A number of studies have demonstrated the crucial role of several fibrogenic cytokines released from immunocytes in initiating the sequence of events leading to fibrosis.

Animal models are useful in providing clues for understanding various human diseases and for exploring new treatments. Although animal models which reproduce all the aspects of SSc are not currently available, bleomycin-induced scleroderma mouse exhibits definite dermal sclerosis mimicking human scleroderma [2]. In this model, features such as definite dermal sclerosis with dermal thickening, pulmonary fibrosis, and the presence of autoantibody in the sera are induced; however, vascular alteration in this model has not been remarked. In this paper, insights into the vascular pathogenesis in bleomycin-induced murine model are discussed.

## 2. Vascular Damages in Human Scleroderma

Vasculopathy in SSc is commonly seen in capillaries and small blood vessels. Raynaud's phenomenon is the common initial sign of SSc in the majority of cases, and digital ulcers, which are refractory and often impair quality of life of patients, are vasculopathies in which intima of vessels can be thickened and the lumen occluded. Vasculopathy in SSc involves several types of cells such as endothelial cells, vascular smooth muscle cells, and pericytes, depending on different phases. Progressive thickening of blood vessel walls with proliferation of vascular intima is the typical feature of SSc [3]. Although the mechanism of intimal proliferation is uncertain, several factors such as chemical influence, virus, stress (e.g., oxidative or ischemia-reperfusion), immune-mediated cytotoxicity, apoptotic process, and antiendothelial cell antibodies (AECAs) are suggested as possible initial triggers. An abnormal response of microvascular endothelial cells to those direct or indirect stimuli may result in vascular injury. Proliferation of vascular smooth muscle cells and pericytes are suggested to lead to the vessel-wall thickening mediated by a Ras-depending manner [4] and occlusive changes by thickened intima. AECAs are frequently detected in sera of patients with SSc [5] and can activate endothelial cells to express cell adhesion molecules which alter leukocyte attachment and lead to endothelial cell damage and apoptosis. Kuwana et al. [6], however, proposed that insufficient vascular repair machinery due to defective vasculogenesis contributes to the microvascular abnormality in SSc. Although circulating concentrations of angiogenic factors are high in SSc, the levels of bone marrow-derived circulating endothelial precursors (CEP) are low [6], especially at late-stage disease [7], suggesting a complex dysregulation of vasculogenesis in SSc. 

 Endothelin-1 (ET-1) is a prototypical endothelial cell-derived product, and endothelial damage leads to increased production of ET-1. Since ET-1 is a vasoconstrictive agent, loss of normal vessel compliance and vasorelaxation may be induced by increased levels of ET-1. In addition, ET-1 promotes fibroblast synthesis of collagen [8]. ET-1 upregulates expression of adhesion molecules, which promote the homing of pathogenic leukocytes to the skin. Further, ET-1 can also induce myofibroblast differentiation in fibroblasts [9]. ET-1 can induce connective tissue growth factor (CTGF), and may mediate the induction of collagen synthesis by activation of CTGF [10]. Circulating ET-1 levels have been observed in patients with diffuse SSc with widespread fibrosis and those with limited SSc and hypertensive disease [11], suggesting that soluble ET-1 levels may be a marker of fibrosis and vascular damage. Thus, ET is suggested to significantly contribute to fibrogenesis, linking between vasculopathy, and fibrosis, and the blockade of ET signaling may lead to the reduction of fibrosis. *In vitro*, SSc fibroblasts synthesized increased amounts of ET-1, and further, bosentan reduced the contractile ability of the SSc fibroblasts [12]. Therefore, a blocking ET-1 might be expected as a benefit in reducing pulmonary fibrosis. Recently, bosentan is demonstrated to reduce the number of newly formation of digital ulcers associated with SSc [13]. Additionally, bosentan may reduce the sclerosis of the skin in a pilot study [14].

 Nitric oxide (NO) is a strong vasodilator and inhibits the biochemical effect of ET-1. However, ET-1 induces inducible NO synthase (iNOS) expression in endothelial cells [15], and iNOS expression is detected in the endothelial cells in the lesional skin of SSc [16]. So far, several reports have shown impaired NO production in SSc [16, 17], which may contribute to the vascular pathogenesis of the arteriolar intimal proliferation in SSc. Thus, an imbalance between vasoconstriction and vasodilatation can lead to ischemia-reperfusion injury, endothelial damage and subsequent increased collagen gene expression *via* hypoxia. Hypoxia induces ECM proteins in cultured fibroblasts, and vascular endothelial growth factor (VEGF) overexpression may be caused in response to chronic hypoxia condition [18].

Reactive oxygen species (ROS) generated during various metabolic and biochemical reactions have multifarious effects that include oxidative damage to DNA. ROS can cause several abnormalities such as endothelial cell damage or enhanced platelet activation, leading to upregulation of the expression of adhesion molecules or secretion of inflammatory or fibrogenic cytokines including platelet-derived growth factor (PDGF) and transforming growth factor-*β* (TGF-*β*); excessive oxidative stress has been implicated in the pathogenesis of scleroderma [19]. Indeed, scleroderma fibroblasts produce ROS constitutively [20]. Other effects of oxygen radicals include the stimulation of skin fibroblast proliferation at low concentrations [21] and the production of increased amounts of collagen [22], suggesting that low oxygen tension may contribute to the increased fibrogenic properties of scleroderma fibroblasts. Furthermore, several of the autoantigens targeted by scleroderma autoantibodies fragment in the presence of ROS and specific metals such as iron or copper [23]. The authors suggest that tissue ischemia generates ROS, which in turn induces the fragmentation of specific autoantigens. On the other hand, oxidative stress transiently induces CCL2 mRNA and protein expression in cultured skin fibroblasts [24], suggesting that ROS may play a regulatory role in inflammation by modulating monocyte chemotactic activity.

## 3. Vascular Changes in Bleomycin-Induced Scleroderma

Bleomycin has a number of biochemical properties, such as blocking the cell cycle at G2, cleaving the single-strand and double-strand DNA, degrading cellular RNAs, production of free radicals, and induction of apoptosis. Bleomycin exerts various effects on skin-constituted cells such as fibroblasts, keratinocytes, and endothelial cells, as well as immunocytes [25]. Bleomycin upregulates gene expression of ECM proteins as well as fibrogenic cytokines such as TGF-*β* and CTGF in cultured human skin fibroblasts [26]. Also, *in vitro* studies showed a dose-dependent stimulation of endothelial cell secretion of collagen synthesis by bleomycin, which was inhibited by the anti-TGF-*β* antibody [27]. 

**Figure 1:**
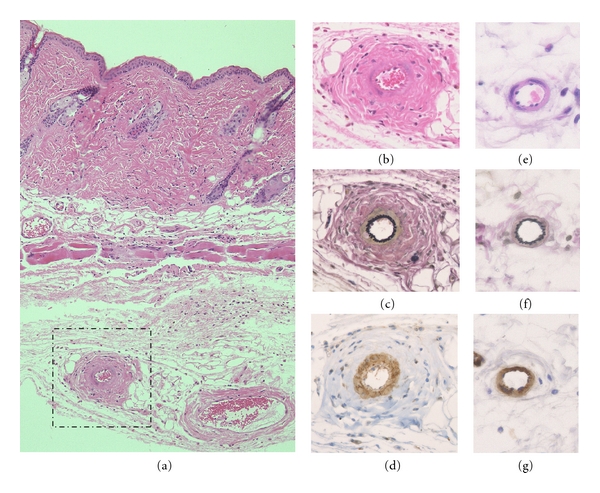
(a) Sclerotic skin induced by bleomycin injection. (b) Close-up view of the vascular lesions showing thickened wall. (c) Elastica van Gieson (EVG) stain showing proliferation of arterial intima. (d) *α*-SMA stain showing proliferation of vascular smooth muscle cells. (e)–(g) Vascular features of control mice treated with PBS were shown (e; H-E, f; EVG, g; *α*-SMA stain).

 Repeated local injections of bleomycin into the back skins induced dermal sclerosis in mice [28–33]. Histopathological examination revealed definite dermal sclerosis characterized by thickened collagen bundles, and the deposition of homogenous materials in the thickened dermis with cellular infiltrates, which mimicked the histologic features of human scleroderma. Dermal thickness gradually increased, up to twofold compared with control PBS injections, with the onset of the sclerosis. Cellular infiltrates were composed of T-cells, monocytes/macrophages, and mast cells, which are supposed to play an important role in the induction of dermal sclerosis. Increased production as well as upregulation of mRNA levels of type I collagen was observed in the bleomycin-treated skin. In the bleomycin-induced scleroderma, *α*-smooth muscle actin- (*α*-SMA-) positive myofibroblasts were observed in the dermis, and gradually increased in tandem with the induction of dermal sclerosis. In addition, significant thickness of vascular wall was also observed in the deep dermis (Figures 1(a) and 1(b)). Elastica van Gieson stain revealed proliferation of vascular intima (Figure 1(c)). Further, *α*-SMA stain suggests proliferation of vascular smooth muscle cells (Figure 1(d)). Those changes were distinct from control PBS-treated mice (Figures 1(e)–1(g)); however, whether the number of capillaries is reduced or not needs further detail investigation.

Recent studies have shown that apoptosis of endothelial cells induces resistance to apoptosis in fibroblasts largely through phosphatidylinositol-3-kinase-dependent mechanisms [33]. Furthermore, fibroblasts exposed to medium conditioned by apoptotic endothelial cells presented myofibroblast changes [34]. By contrast, cultured scleroderma fibroblasts were resistant to Fas-induced apoptosis [35, 36]. Although the effect of TGF-*β* on apoptosis differs according to cell type, stage of maturation, and other factors, TGF-*β*1 may play a role in inducing apoptosis-resistant fibroblast populations in SSc [36]. In scleroderma fibroblasts, Bcl-2 level was significantly higher, whereas the Bax level significantly decreased [36]. In primary pulmonary endothelial cells, bleomycin initiates apoptosis *via* the extrinsic pathway [37]. Also, involvement of the extrinsic apoptotic process in the bleomycin-induced scleroderma model has been investigated. DNA fragmentation revealed laddering of the bleomycin-treated skin, and increased expression of Fas and FasL was detected in the lesional skin. mRNA expression as well as activity of caspase-3 was also enhanced after bleomycin treatment. Administration of neutralizing anti-FasL antibody together with local bleomycin treatment reduced the development of dermal sclerosis, in association with the reduction of TUNEL-positive mononuclear cells and with the blockade of apoptosis. Caspase-3 activity was also significantly reduced after anti-FasL treatment. Excessive apoptosis, which cannot be treated by macrophages, may induce proinflammatory cytokines such as tumor necrosis factor-*α* (TNF-*α*) or interferons (IFNs), and play a triggering role in the pathogenesis of bleomycin-induced scleroderma. In the bleomycin model, TUNEL-positivity was prominently detected on keratinocytes and infiltrating mononuclear cells, but not endothelial cell and fibroblasts in the sclerotic skin [38]. 

Vascular injury causes endothelial cell activation, dysfunction and altered capillary permeability as a primary event. These are followed by increased expression of adhesion molecules leading to mononuclear cell infiltrates in the skin. Cellular adhesion molecules (CAMs) are suspected of being responsible for the homing of pathologic inflammatory cells to the skin and are involved between immune cells, fibroblasts, endothelial cells, and ECM in the lesional skin of scleroderma. *In vitro*, bleomycin directly induces E-selectin expression in endothelial cells through activation and nuclear translocation of NF-*κ*B/Rel [39]. Also, *in vivo* studies show that intradermal injections of bleomycin into the normal human skin upregulate the expression of intercellular adhesion molecule-1 (ICAM-1) and E-selectin [40]. Those molecules play an important role in activation and migration of lymphocytes across the endothelium and basement membranes and in adherence to target tissues. The adhesion step may be important to the development of the initial pathologic changes of bleomycin-induced scleroderma.

## 4. Conclusion

In this paper, we have made a focus on vascular features of a bleomycin-induced murine scleroderma. Better understandings of the pathophysiology of collagen vascular disease in scleroderma are expected to contribute to the novel therapies specifically targeting vasculopathy of SSc.
